# Supplementary material for: Nicotine Dependence in a Banned Market: Biomarker Evidence from E-Cigarette Users in São Paulo, Brazil
Source: Int J Environ Res Public Health. 2025 Jun 19;22(6):960. doi: 10.3390/ijerph22060960 (PMC12193091; doi:10.3390/ijerph22060960)
Supplement: Supplementary file 1 [file ijerph-22-00960-s001.zip › Supplemental material_Materials and Methods_LCMSMS analysis.pdf]

Supplemental Material

Materials and Methods

Nicotine and Cotinine Determination in Oral Fluid

Mobile phase was constituted of ultrapure water, 0.002% formic acid and 0,002 mol/L ammonium formate (solvent A) and methanol, 0.002% formic acid and 0,002 mol/L ammonium formate (solvent B); sample manager wash was methanol/acetonitrile/isopropanol/ ultrapure water (25:25:25:25, v:v:v:v) flow rate was 0.3 mL/min; gradient is detailed on Table S6. Electrospray ionization (ESI)-MS/MS detection was performed in multiple reaction monitoring (MRM) and argon gas was used in the collision step. The instrument conditions for each analyte were individually optimized and Table S7 exhibit the parameters, and Table S8, parameters and settings LC/MS-MS.

Table S6. Mobile phase gradient for the chromatographic separation in ultra-high pressure liquid chromatography

	Time (minutes)	A (%)	B (%)
1	2	97	3
2	4	0	100
3	2	97	3

Total time of chromatographic separation: 6 minutes

Table S7. Analytes cotinine and nicotine, and cotinine-d3 internal standard (IS) their retention times, precursor ion (m/z), product ions, MRM conditions optimized for the LC-MS/MS analysis in oral fluid

Analyte	Retention time (minutes)	Precursor ion (m/z)	Product ions (m/z)	Q1 Pre bias (V)	CE (eV)	Q3 Pre bias (V)
Cotinine	2.624	177	**80**	-32	-20	-30
			98	-32	-20	-30
			146	-32	-16	-30
Cotinine-d3 (IS)	2.610	180	**80**	-15	-18	-17
			101	-15	-14	-13
Nicotine	2.001	163	**130**	-20	-25	-20
			117	-20	-25	-20

IS = Internal Standard; CE = Collision Energy

Q1 Pre Bias = voltage promotes the ionization of the precursor ion; Q3 Pre Bias = voltage promotes the ionization of the product ion

In bold are displayed the quantifier ions.

Table S8. Parameters and settings LC/MS-MS

Item	Setting
Pump system flow	0.3 mL/min
Oven temperature	45 °C
Nebulizing gas flow	3.0 L/min
Drying gas flow	10 L/min
Heating gas flow	10 L/min
Interface	ESI
Interface voltage	4.0 kV
Interface temperature	300°C
Desolvation temperature	526°C
DL temperature	250°C
Heat block temperature	400°C
Detector voltage	1.92 kV

ESI = Electrospray Ionization; DL = Desolvation Line

The method was validated considering limits of detection (LODs), limits of quantification (LOQs), calibration curves, intraday and interday precision and accuracy, dilution integrity, carryover, and matrix effects. The validation followed international guidelines and recommendations for forensic toxicology [1,2].

The LOD was defined as the concentration yielding a signal-to-noise ratio greater than 3. The LOQ was the lowest concentration that could be quantified with acceptable precision and accuracy, defined by acceptance criteria of ±15% for both precision (relative standard deviation, RSD) and accuracy bias.

A calibration curve was obtained by extraction of oral fluid spiked with defined concentrations. Each concentration was analyzed in five replicates. We evaluated heteroscedasticity by application of the F-test for each analyte [3]. When present, weighting factors were applied to improve linearity.

The linearity study was performed by analyzing aliquots of oral fluid containing nicotine and cotinine in six replicates at the following concentrations for each analyte: Cotinine: 1, 10, 100, 200, 500, 1000 and 2000 ng/mL, and nicotine: 5, 20, 100, 200, 500, 1000 and 2000 ng/mL.

Precision and accuracy were assessed by analyzing oral fluid samples at concentrations of 25, 75, and 1500 ng/mL for each analyte across three consecutive days, with six replicates per day. Precision (RSD) was calculated for both intraday and interday variability. Accuracy was expressed as percent bias, calculated as: (mean measured concentration / nominal concentration) × 100.

Dilution integrity is essential when analyzing oral fluid. For example: biological samples volume is lower than the minimum validated by research or the first result exam is higher than the upper point calibration curve. To ensure the reliability of the results, the dilution integrity was validated for 5000, 10000, and 15000 ng/mL. The analyses were performed in triplicate for each concentration. Accuracy and precision were required to be within the set criteria (within ±20%) and results are in Table S9.

Carryover was tested by analyzing a blank sample immediately after a high-concentration sample (2000 ng/mL for both analytes). No carryover was detected.

The matrix effect was evaluated by comparing peak areas of two sample sets: (A) quality control samples spiked in acetonitrile, and (B) quality control samples spiked in oral fluid. Each condition was tested in five replicates. The matrix effect was assessed by comparing the average peak areas between the two matrices.

The LOD and LOQ were confirmed as the lowest concentrations within the linear range for which precision and accuracy met the acceptance criteria.

The method demonstrated good linearity for all analytes ($r^2 > 0.99$). Cotinine calibration curves were homoscedastic and did not require weighting. Nicotine exhibited heteroscedasticity, for which weighted regression was applied. No matrix effects or carryover were observed. Precision and accuracy were maintained after sample dilution, with all results within ±20% RSD, as shown in Table S10.

Table S9. Precision and accuracy for cotinine and nicotine after dilution integrity analysis

**Analyte**	**Precision**			**Accuracy**		
	**1:5**	**1:10**	**1:15**	**1:5**	**1:10**	**1:15**
Cotinine	6.2	5.8	7.5	11.1	6.6	8.1
Nicotine	10.2	8.6	5.2	5.5	5.1	9.8

Table S10. Results for linearity (calibration curve, regression equations, coefficient of determination – r^2, weighting factor for cotinine and nicotine), precision (expressed as relative standard deviation -%) and accuracy (expressed as percentage -%) obtained during the validation. Precision and accuracy results are expressed for the low-quality controls (LQC, 25 ng/mL), medium-quality controls (MQC, 75 ng/mL) and high quality controls (HQC, 1000 ng/mL)

Analyte	Linearity				Precision			Accuracy		
	Calibration curve (ng/mL)	Regression equations	R^2	Weighting factor	LQC (%)	MQC (%)	HQC (%)	LQC (%)	MQC (%)	HQC (%)
Cotinine	1, 10, 100, 200, 500, 1000, 2000	y = 0.01501816x + 0.002487788	0.998	None	5.1	4.8	10.5	7.9	3.4	9.4
Nicotine	5, 20, 100, 200, 500, 1000, 2000	y = 0.004863125x + 0.01613596	0.995	1/A^2	8.8	2.7	6.7	11.7	13.8	11.5

References

1. Peters FT, Drummer OH, Musshoff F. Validation of new methods. *Forensic Sci Int*. 2007;165(2-3):216-24.
2. LeBeau MA. ANSI/ASB standard 036 for method validation in forensic toxicology has replaced SWGTOX's version. *J Anal Toxicol*. 2020;44(4):414. https://doi.org/10.1093/jat/bkz115
3. Almeida AM, Castel-Branco MM, Falcão AC. Linear regression for calibration lines revisited: weighting schemes for bioanalytical methods. *J Chromatogr B*. 2002;774:215-22. https://doi.org/10.1016/S1570-0232(02)00244-1)
