# Supplementary material for: Nicotine Dependence in a Banned Market: Biomarker Evidence from E-Cigarette Users in São Paulo, Brazil
Source: Int J Environ Res Public Health. 2025 Jun 19;22(6):960. doi: 10.3390/ijerph22060960 (PMC12193091; doi:10.3390/ijerph22060960)
Supplement: Supplementary file 1 [file ijerph-22-00960-s001.zip › Supplemental material_Technicians.pdf]

List of technicians from the Health Surveillance Center of the State Department of Health of São Paulo

São Paulo city
Ailton Catreus de Freitas
Antonia da Luz Oliveira
Elaine Cristine D'Amico
Emma Valeria Marchiori
Geralda Luciene Costa da Silva
Lourinaldo Cordeiro Alves
Luzia Maria Belo
Nea Miwa Kashiwagi
Nilson Betarelli
Rita de Cassia Dias Carreira Bacoccini
Rosemairy Norye Inamine
Rubens José Mario Junior
Susi Maria Cortes Quevedo

Santo André
Camila Dias da Cruz
Claudia Regina Polidoro
Daniele Cristine Assi
Edilson César Dias
Edna Correa Clares
Elaine Atanes de Jesus
Fernanda Prenholato Gouveia
Flávio Pereira
Iris Silvério da Silva Bento
Kátia Regina Soares dos Santos
Loyde Rodrigues Lima Machado
Maria Aparecida de Oliveira D'onofrio
Marli Yukie Arioshi Sanches
Patricia Bezerra da Silva
Sonia Oliveira Barbosa Muraro
Telma Cristina Souza Silva

Ribeirão Preto
Ana Estela Belon Fernandes de Siqueira
Jucelia Alves da Silva
Márcia Aparecida Thomaz
Monica Aparecida Lourenço

Campinas
Carla Regina Menezes de Pompeo
Fátima Rodrigues Torres

José Roberto dos Santos
Marcia Elena Simal Fante
Maria das Dores Ribeiro Ilha

Santos
Eliane Maria Mancilha
Elias Carlos Daccache
Giuliana Azevedo Bono
Márcia Regina Cauchioli

Taubaté / Campos do Jordão
Andrea Maria G. V. Consolino
Antonia Benedita O. Cardoso
Darcy de Paula F. da Silva
Heloisa Moura de Paula L. Zanotta
Kamilla Borges de Souza
Maria de Fatima F. Teixeira
Maria do Carmo Toledo
Sandra Justen
Selma A. Machado Pelogia
