# Supplementary material for: Nicotine Dependence in a Banned Market: Biomarker Evidence from E-Cigarette Users in São Paulo, Brazil
Source: Int J Environ Res Public Health. 2025 Jun 19;22(6):960. doi: 10.3390/ijerph22060960 (PMC12193091; doi:10.3390/ijerph22060960)
Supplement: Supplementary file 1 [file ijerph-22-00960-s001.zip › Supplemental Tables_IJERPH 2025.pdf]

Supplemental Table S1. Results for multivariate linear regression for nicotine analysis

	Initial model		Final model	
	Adjusted coefficient (95% CI)	p	Adjusted coefficient (95% CI)	p
Gender, male (ref. female)	43.6 (-69.9–157.0)	0.450	-	-
Age range (ref. ≤25 years)		0.262	-	-
26–35 years	68.8 (-65.5–203.0)	0.314		-
36–45 years	-74.7 (-243.6–94.3)	0.385	-	-
≥46 years	144.8 (-120.5–410.1)	0.284	-	-
Body mass index	1.3 (-11.5–14.1)	0.840	-	-
Years spend on education (ref. ≤9 years)		0.797	-	-
10–15 years	100.9 (-201.0–402.9)	0.511	-	-
≥16 years	85.8 (-215.9–387.5)	0.576		-
Former smoker	47.6 (-67.7–162.8)	0.417	-	-
Presence of anxiety or depression	70.9 (-52.1–193.9)	0.257	-	-
Consumption duration (ref. ≤1 year)		0.200		-
2–3 years	-52.1 (-173.0–68.8)	0.397	-	-
≥4 years	85.9 (-79.8–251.6)	0.309	-	-
Recharge/purchase frequency (ref. occasional)		0.793		-
Daily	39.1 (-169.4–247.5)	0.713	-	-
Weekly	67.6 (-101.1–236.3)	0.431	-	-
Every two weeks	-91.4 (-487.7–304.9)	0.650	-	-
Monthly	-6.4 (-167.1–154.3)	0.937	-	-
Knowledge of nicotine content (ref. no knowledge)		0.941		-
Aware of nicotine content	-18.2 (-142.4–106.0)	0.773	-	-
Not aware of nicotine content	16.4 (-191.6–224.3)	0.877	-	-
Perception of addiction (ref. none)		0.746		-
Mild	51.9 (-99.5–203.3)	0.501	-	-
Moderate	-32.3 (-196.9–132.4)	0.700	-	-
Severe	63.4 (-122.5–249.3)	0.503	-	-
Unsure	-86.9 (-490.8–317.1)	0.673	-	-
Last consumption (ref. 48 hours ago)		0.337		0.004
30 minutes ago	217.5 (38.7–396.4)	0.017	277.4 (145.7–409.1)	<0.001

31–60 minutes ago	167.2 (-42.8–377.2)	0.118	196.4 (24.0–368.8)	0.026
61–90 minutes ago	21.0 (-295.8–337.8)	0.896	107.8 (-165.8–381.4)	0.439
90–120 minutes ago	99.9 (-274.7–474.4)	0.600	142.4 (-194.6–479.3)	0.407
>2 hours ago	144.1 (-146.4–434.7)	0.330	152.0 (-106.1–410.1)	0.247
~12 hours ago	53.9 (-265.0–372.8)	0.740	72.8 (-200.8–346.4)	0.601
~24 hours ago	48.2 (-193.6–290.1)	0.695	50.3 (-162.2–262.8)	0.642
**N**	332		368	
**R^2**	9.5%		5.6%	
**Adjusted R^2**	1.1%		3.7%	

95% CI, 95% confidence interval; N, total number of participants considered in the analysis.

Supplemental Table S2. Results for multivariate linear regression for cotinine analysis

	Initial model		Final model	
	Adjusted coefficient (95% CI)	p	Adjusted coefficient (95% CI)	p
Gender, male (ref. female)	11.5 (-9.3–32.3)	0.277	-	-
Age	0.8 (-0.4–2.0)	0.210	-	-
Body mass index	-0.3 (-2.6–2.0)	0.803	-	-
Years spend on education (ref. ≤9 years)		0.507		-
10–15 years	23.7 (-31.9–79.3)	0.402	-	-
≥16 years	13.6 (-42.0–69.2)	0.630	-	-
Former smoker	3.6 (-17.4–24.7)	0.735	-	-
Presence of anxiety or depression	22.0 (-0.7–44.6)	0.057	-	-
Consumption duration (ref. ≤1 year)		0.786		-
2–3 years	-0.7 (-23.0–21.5)	0.947	-	-
≥4 years	-10.0 (-40.5–20.5)	0.519	-	-
Recharge/purchase frequency (ref. occasional)		0.010		0.008
Daily	27.6 (-10.8–66.0)	0.158	30.4 (-5.2–66.0)	0.094
Weekly	35.0 (4.0–65.9)	0.027	30.3 (0.5–60.1)	0.047
Every two weeks	123.1 (50.3–196.0)	0.001	128.9 (57.2–200.6)	<0.001
Monthly	15.7 (-13.8–45.2)	0.295	15.6 (-12.7–43.9)	0.278
Knowledge of nicotine content (ref. no knowledge)		0.868		-
Aware of nicotine content	5.6 (-17.0–28.3)	0.626	-	-
Not aware of nicotine content	5.4 (-32.8–43.7)	0.780	-	-
Perception of addiction (ref. none)		0.026		0.017
Mild	27.0 (-0.9–54.8)	0.058	26.0 (-0.8–52.7)	0.057
Moderate	34.5 (4.2–64.7)	0.026	32.9 (5.5–60.2)	0.019
Severe	55.3 (21.0–89.5)	0.002	51.3 (21.3–81.3)	0.001
Unsure	-12.3 (-86.6–62.0)	0.744	20.3 (-48.4–89.1)	0.561
Last consumption (ref. 48 hours ago)		0.004		0.001
30 minutes ago	61.9 (29.3–94.4)	<0.001	63.3 (32.9–93.7)	<0.001
31–60 minutes ago	62.9 (24.3–101.5)	0.001	61.0 (24.4–97.6)	0.001
61–90 minutes ago	3.1 (-55.0–61.2)	0.916	4.2 (-51.7–60.1)	0.882

90–120 minutes ago	23.4 (-45.5–92.2)	0.505	23.4 (-44.5–91.3)	0.499
>2 hours ago	26.6 (-26.7–79.9)	0.327	25.3 (-26.5–77.1)	0.338
~12 hours ago	21.2 (-37.3–79.6)	0.477	22.4 (-34.5–79.4)	0.439
~24 hours ago	18.1 (-26.5–62.6)	0.426	10.4 (-32.2–53.0)	0.632
**N**	332		362	
**R^2**	26.7%		23.5%	
**Adjusted R^2**	20.4%		20.2%	

95% CI, 95% confidence interval; N, total number of participants considered in the analysis.

Supplemental Table S3. Nicotine and cotinine concentrations in oral fluid according to devices characteristics products and about nicotine

	N	Nicotine levels (ng/mL)			Cotinine levels (ng/mL)		
		**Mean ± SD**	**Median (IQR)**	**p**	**Mean ± SD**	**Median (IQR)**	**p**
Knowledge of nicotine content				<0.001			<0.001
No knowledge	206	184.69 ± 477.83§	27.00 (5.00–139.75)		64.77 ± 93.11§	20.00 (2.00–84.00)	
Aware of nicotine content	138	227.43 ± 459.30ǂ	66.00 (16.75–182.75)		104.03 ± 105.89ǂ	84.00 (16.75–147.25)	
Not aware of nicotine content	29	121.45 ± 348.75§	5.00 (0.00–76.00)		40.03 ± 65.81§	5.00 (0.00–51.00)	
Nicotine form				<0.001			<0.001
Nicotine salt	93	264.76 ± 513.14ǂ	87.00 (28.00–209.50)		122.86 ± 119.20ǂ	88.00 (32.50–175.50)	
Nicotine free-base	40	129.60 ± 298.32§	30.50 (0.00–87.00)		65.78 ± 71.59§	42.00 (3.25–105.75)	
Both nicotine salt and free-base	6	366.17 ± 389.72	293.00 (21.75–688.00)		109.50 ± 97.44	97.00 (14.00–199.75)	
No knowledge	208	181.01 ± 474.75§	27.00 (5.00–139.00)		62.98 ± 89.44§	20.00 (2.00–84.00)	
No nicotine	26	126.88 ± 367.04§	5.00 (0.00–75.00)		40.27 ± 67.10§	6.50 (1.50–49.00)	
Nicotine salt concentration				0.221			0.148
20 mg/mL	17	67.35 ± 59.54	43.00 (24.50–102.00)		93.35 ± 97.73	72.00 (21.00–155.00)	
35 mg/mL	23	335.00 ± 702.17	99.00 (44.00–209.00)		119.09 ± 99.47	103.00 (39.00–175.00)	
50 mg/mL	51	321.24 ± 501.78	127.00 (30.00–459.00)		140.45 ± 128.01	98.00 (44.00–200.00)	
Unknown	8	198.38 ± 287.55	98.00 (9.75–315.75)		74.25 ± 128.75	21.00 (13.25–66.50)	
Nicotine free-base concentration				0.697			0.607
I don't know	2	40.50 ± 57.28	-		59.50 ± 81.32	-	
3 mg/mL	27	184.00 ± 363.40	46.00 (0.00–134.00)		74.85 ± 73.39	73.00 (6.00–117.00)	

6 mg/mL	15	153.53 ± 266.97	25.00 (6.00–87.00)	64.00 ± 81.69	32.00 (2.00–103.00)
12 mg/mL	1	29.00 ± 0.00	-	180.00 ± 0.00	-
>20 mg/mL	1	0.00 ± 0.00	-	8.00 ± 0.00	-

p values are descriptive levels for Kruskal-Wallis test.

ǂ and § indicate distinct means according to Dunn-Bonferroni multiple comparisons.

Supplemental Table S4. Sociodemographic data, smoking history, e-cig use patterns, perception of addiction, and cotinine concentrations according to nicotine concentrations

	Overall	Nicotine levels		p*
		**<400 ng/mL (N=327; 87,0%)**	**≥400 ng/mL (N=49; 13,0%)**	
Gender, n (%)	N=376	N=327	N=49	
Male	195 (51.9)	166 (50.8)	29 (59.2)	
Female	181 (48.1)	161 (49.2)	20 (40.8)	
Age, years	N=376	N=327	N=49	0.828[a]
Mean ± SD	27.5 ± 9.1	27.6 ± 9.2	27.1 ± 8.9	
Median (IQR)	25.0 (20.3–32.0)	25.0 (21.0–32.0)	25.0 (20.0–32.0)	
Smoking history, n (%)	N=362	N=316	N=46	0.273[b]
Former smoker	154 (42.5)	131 (41.5)	23 (50.0)	
Naïve smoker	208 (57.5)	185 (58.5)	23 (50.0)	
Consumption duration, n (%)	N=369	N=320	N=49	0.184[b]
≤1 year	140 (37.7)	119 (37.0)	21 (42.9)	
2–3 years	164 (44.2)	148 (46.0)	16 (32.7)	
≥4 years	67 (18.1)	55 (17.1)	12 (24.5)	
Last consumption, n (%)	N=371	N=322	N=49	0.051[c]
30 minutes ago	184 (49.6)	150 (46.6)	34 (69.4)	
31–60 minutes ago	48 (12.9)	41 (12.7)	7 (14.3)	
61–90 minutes ago	13 (3.5)	11 (3.4)	2 (4.1)	
90–120 minutes ago	8 (2.2)	7 (2.2)	1 (2.0)	
>2 hours ago	15 (4.0)	14 (4.3)	1 (2.0)	
~12 hours ago	13 (3.5)	12 (3.7)	1 (2.0)	
~24 hours ago	25 (6.7)	23 (7.1)	2 (4.1)	
~48 hours ago	62 (16.7)	61 (18.9)	1 (2.0)	
Last week	2 (0.5)	2 (0.6)	0 (0.0)	
Last month	1 (0.3)	1 (0.3)	0 (0.0)	
Recharge/purchase frequency, n (%)	N=369	N=320	N=49	0.131[b]
Daily	53 (14.4)	42 (13.1)	11 (22.4)	
Weekly	118 (32.0)	100 (31.3)	18 (36.7)	
Every two weeks	7 (1.9)	6 (1.9)	1 (2.0)	
Monthly	116 (31.4)	101 (31.6)	15 (30.6)	

Occasional	75 (20.3)	71 (22.2)	4 (8.2)	
Nicotine form, n (%)	N=373	N=324	N=49	
Nicotine salt	99 (26.5)	80 (24.7)	19 (38.8)	0.037[b]
Nicotine free-base	46 (12.3)	39 (12.0)	7 (14.3)	0.655[b]
No knowledge	208 (55.8)	184 (56.8)	24 (49.0)	0.305[b]
No nicotine	26 (7.0)	24 (7.4)	2 (4.1)	0.553[c]
Perception of addiction, n (%)	N=374	N=325	N=49	0.083[b]
None	139 (37.2)	125 (38.5)	14 (28.6)	
Mild	79 (21.1)	69 (21.2)	10 (20.4)	
Moderate	82 (21.9)	72 (22.2)	10 (20.4)	
Severe	65 (17.4)	50 (15.4)	15 (30.6)	
Unsure	9 (2.4)	9 (2.8)	0 (0.0)	
Cotinine, ng/mL	N=376	N=327	N=49	<0.001[a]
Mean ± SD	76.82 ± 98.25	65.18 ± 89.84	154.55 ± 116.22	

N, total number of participants considered in the analysis; n, number of participants within a subgroup; SD, standard deviation; IQR, interquartile range.

* p values are descriptive levels for Mann-Whitney test[a], Chi-Square test[b], and Fisher exact test[c].

Supplemental Table S5. Participants' perception of health and social impact, knowledge of risks and environmental consequences, perception of addiction, exposure to risk, attempts to quit vaping, future perspectives on e-cigarette use, and opinion on government regulations according to smoking history

	Overall	Smoking history		p*
		Naïve smokers	Former smokers	
Perception of impact on health status, n (%)	N=387	N=222	N=165	<0.001[a]
No change	247 (63.8)	158 (71.2)	89 (53.9)	
Health deterioration	97 (25.1)	54 (24.3)	43 (26.1)	
Health improvement	43 (11.1)	10 (4.5)	33 (20.0)	
Social impact, n (%)	N=400	N=229	N=171	0.136[a]
No change	318 (79.5)	190 (83.0)	128 (74.9)	
Positively affected	61 (15.3)	29 (12.7)	32 (18.7)	
Negatively affected	21 (5.3)	10 (4.4)	11 (6.4)	
Knowledge of risks, n (%)	N=399	N=229	N=170	0.042[b]
Yes	308 (77.2)	188 (82.1)	120 (70.6)	
No	34 (8.5)	16 (7.0)	18 (10.6)	
Some knowledge	54 (13.5)	24 (10.5)	30 (17.6)	
No risks	3 (0.8)	1 (0.4)	2 (1.2)	
Knowledge of environmental consequences, n (%)	N=401	N=228	N=173	0.673[a]
Yes	173 (43.1)	100 (43.9)	73 (42.2)	
No	157 (39.2)	91 (39.9)	66 (38.2)	
Uncertain	71 (17.7)	37 (16.2)	34 (19.7)	
Perception of addiction, n (%)	N=401	N=228	N=173	<0.001[a]
None	150 (37.4)	107 (46.9)	43 (24.9)	
Mild	86 (21.4)	50 (21.9)	36 (20.8)	
Moderate	91 (22.7)	37 (16.2)	54 (31.2)	
Severe	66 (16.5)	30 (13.2)	36 (20.8)	
Unsure	8 (2.0)	4 (1.8)	4 (2.3)	
Exposure to risk, n (%)	N=401	N=228	N=173	0.845[b]
Yes	17 (4.2)	11 (4.8)	6 (3.5)	
No	377 (94.0)	212 (93.0)	165 (95.4)	
Uncertain	6 (1.5)	4 (1.8)	2 (1.2)	
No answer	1 (0.2)	1 (0.4)	0 (0.0)	

Attempts to quit vaping, n (%)	N=401	N=228	N=173	0.315[a]
Yes	272 (67.8)	150 (65.8)	122 (70.5)	
No	129 (32.2)	78 (34.2)	51 (29.5)	
Future perspectives on e-cigarette use, n (%)	N=402	N=229	N=173	0.222[a]
Will continue to use	112 (27.9)	57 (24.9)	55 (31.8)	
Will quit vaping	176 (43.8)	101 (44.1)	75 (43.4)	
Maybe will quit vaping	114 (28.4)	71 (31.0)	43 (24.9)	
Opinion on government regulations, n (%)	N=401	N=229	N=172	0.015[a]
Agree	94 (23.4)	59 (25.8)	35 (20.3)	
Disagree	244 (60.8)	126 (55.0)	118 (68.6)	
Unsure	63 (15.7)	44 (19.2)	19 (11.0)	

N, total number of participants considered in the analysis; n, number of participants within a subgroup.

* p values are descriptive levels for Chi-Square test[a], Fisher exact test[b], and Mann-Whitney test[c].
